# Breathlessness across generations: results from the RHINESSA generation study

**DOI:** 10.1136/thoraxjnl-2021-217271

**Published:** 2021-06-14

**Authors:** Magnus Ekström, Ane Johannessen, Michael J Abramson, Bryndis Benediktsdottir, Karl Franklin, Thorarinn Gislason, Francisco Gómez Real, Mathias Holm, Christer Janson, Rain Jogi, Adrian Lowe, Andrei Malinovschi, Jesús Martínez-Moratalla, Anna Oudin, José Luis Sánchez-Ramos, Vivi Schlünssen, Cecilie Svanes

**Affiliations:** 1 Department of Respiratory Medicine and Allergology, Lund University, Lund, Sweden; 2 Department of Global Public Health and Primary Care, University of Bergen, Bergen, Norway; 3 School of Public Health & Preventive Medicine, Monash University, Melbourne, Victoria, Australia; 4 Department of Sleep, Landspitali University Hospital, Reykjavik, Iceland; 5 Faculty of Medicine, University of Iceland, Reykjavik, Iceland; 6 Department of Surgery and Perioperative Sciences, Umeå University, Umeå, Sweden; 7 Department of Clinical Science, University of Bergen, Bergen, Norway; 8 Department of Obstetrics and Gynecology, Haukeland University Hospital, Bergen, Norway; 9 Occupational and Environmental Medicine, University of Gothenburg, Gothenburg, Sweden; 10 Department of Medical Sciences, Uppsala University, Uppsala, Sweden; 11 The Lung Clinic, Tartu University Hospital, Tartu, Estonia; 12 Melbourne School of Population and Global Health, The University of Melbourne, Melbourne, Victoria, Australia; 13 Servicio de Pneumologıa del Complejo Hospitalario Universitario de Albacete (CHUA), Servicio de Salud de Castilla-La Mancha (SESCAM), Albacete, Spain; 14 Department of Public Health and Clinical Medicine, Sustainable Health, Umeå University, Umeå, Sweden; 15 Nursing Department, University of Huelva, Huelva, Spain; 16 Department of Public Health, Environment, Work and Health, Danish Ramazzini Center, Aarhus University, Aarhus, Denmark; 17 Department of Public Health, Department of Public Health, Environment, Work and Health, Danish Ramazzini Center, Copenhagen, Denmark; 18 Department of Occupational Medicine, Haukeland University Hospital, Bergen, Norway

**Keywords:** perception of asthma/breathlessness, asthma, clinical epidemiology

## Abstract

**Background:**

Breathlessness is a major cause of suffering and disability globally. The symptom relates to multiple factors including asthma and lung function, which are influenced by hereditary factors. No study has evaluated potential inheritance of breathlessness itself across generations.

**Methods:**

We analysed the association between breathlessness in parents and their offspring in the Respiratory Health in Northern Europe, Spain and Australia generation study. Data on parents and offspring aged ≥18 years across 10 study centres in seven countries included demographics, self-reported breathlessness, asthma, depression, smoking, physical activity level, measured Body Mass Index and spirometry. Data were analysed using multivariable logistic regression accounting for clustering within centres and between siblings.

**Results:**

A total of 1720 parents (mean age at assessment 36 years, 55% mothers) and 2476 offspring (mean 30 years, 55% daughters) were included. Breathlessness was reported by 809 (32.7%) parents and 363 (14.7%) offspring. Factors independently associated with breathlessness in parents and offspring included obesity, current smoking, asthma, depression, lower lung function and female sex. After adjusting for potential confounders, parents with breathlessness were more likely to have offspring with breathlessness, adjusted OR 1.8 (95% CI 1.1 to 2.9). The association was not modified by sex of the parent or offspring.

**Conclusion:**

Parents with breathlessness were more likely to have children who developed breathlessness, after adjusting for asthma, lung function, obesity, smoking, depression and female sex in both generations. The hereditary components of breathlessness need to be further explored.

**Key messages**

**What is the key question?**

- Does breathlessness cluster across generations?

**What is the bottom line?**

- Breathlessness was nearly doubled in offspring of parents with breathlessness, even when adjusting for factors associated with breathlessness in both generations (obesity, smoking, depression, asthma, lower lung function and female sex).

**Why read on?**

- This is the first large population study evaluating breathlessness across generations which supports potential hereditability of breathlessness.

## Introduction

Breathlessness related to daily activities is common in many populations, affecting 15%–45% of middle-aged and older people in the community.^1–3^ Physical disability due to activity-related breathlessness, measured using the modified Medical Research Council (mMRC) scale,^4^ is associated with major adverse health outcomes including impaired quality of life^1 5 6^ and earlier death.^7 8^

Breathlessness in the population is related to multiple factors including cardiorespiratory disease, obesity, deconditioning, mental state, occupational and environmental exposures, and early life factors.^1–3 9–12^ Ventilatory capacity, assessed using FEV_1_ and FVC, is related to breathlessness. This could explain why breathlessness is more prevalent in women, having smaller lungs and airways compared with men.^13–15^ Furthermore, airflow obstruction is strongly related to increased breathlessness,^1 2 12–15^ with asthma being an especially prevalent risk factor at younger ages.^16^

While heritability in asthma^17–19^ and lung function^20–22^ is well established, it is unknown whether breathlessness clusters across generations. No study has evaluated whether parents with breathlessness are more likely to have offspring with breathlessness or the potential factors underpinning any such association. Clustering of breathlessness across the generations is conceivable, as several determinants of breathlessness are known to be at least partly hereditary, including level of lung function,^20 21^ physical activity,^23^ obesity and body fat composition, and asthma.^17–19^

The primary aim of this study was to evaluate whether parents’ breathlessness was a risk factor for offspring breathlessness, when accounting known risk factors for breathlessness in both generations. Second, we aimed to explore potential sex-specific patterns by evaluating if associations in breathlessness across generations were modified by the sex of the parent and/or the offspring.

## Methods

### Study design and population

This was an analysis within the Respiratory Health in Northern Europe, Spain and Australia (RHINESSA) generation study.^24^ Parents were identified from the European Community Respiratory Health Survey (ECRHS, www.ecrhs.org). In 10 ECRHS study centres, data on their offspring were obtained for the RHINESSA study (www.rhinessa.net). The analysis database comprised one parent (mother or father) and one or more offspring for each parent. This study is reported in accordance with the Strengthening the Reporting of Observational Studies in Epidemiology guidelines.^25^

#### Parents

The ECRHS study surveyed population-based random samples of adults aged 20–44 years in 1992 (~3000 per research centre) in 56 study centres across 25 countries.^20 21^ This study includes the cohort from 10 study centres in Northern Europe, Spain and Australia with clinical examinations including spirometry. These 10 centres were Aarhus (Denmark); Bergen (Norway); Gothenburg, Uppsala and Umeå (Sweden); Reykjavik (Iceland); Tartu (Estonia); Huelva and Albacete (Spain); and Melbourne (Australia). The remaining 46 centres did not examine the participants’ offspring and were consequently not part of the RHINESSA generation study.

#### Offspring

All adult offspring (≥18 years) of parents from the following ten ECRHS centres were invited to complete questionnaires and participate in clinical examination including spirometry in 2013–2015.^24^ Spirometry was performed using standardised protocols similar to those used for investigation of their parents.^24^ The questionnaires were web-based in all centres except for the Swedish centres, where postal questionnaires were used. In total, 33.5% of the invited adult offspring participated in the study, with response rates varying across centres from 18.6% in Tartu to 73.7% in Melbourne.^26^

The current analysis is based on this adult offspring study of men and women aged ≥18 years with physiological data including spirometry. Exclusion criteria for the present analysis were inability to walk for other reasons than breathlessness (as most breathlessness is related to physical activity) and missing data on breathlessness in parents or offspring.

### Assessments and definitions

Breathlessness was defined similarly in parents and offspring as the self-report of any of the following symptoms during the last 12 months: (1) breathlessness when having wheezing and whistling in the chest, (2) attack of shortness of breath when at rest, (3) attack of shortness of breath following strenuous activity or (4) being woken by an attack of shortness of breath. Breathlessness and other factors were assessed at the first available data point for parents (ECHRS I) and in offspring (RHINESSA). In parents, data were also available on breathlessness on the mMRC breathlessness scale.^4^

Data were obtained for both parents and offspring on age; sex; study centre; self-reported asthma diagnosed by a physician; history of depression; smoking status (never, former or current smoker); pack-years of smoking; level of highest completed education (primary, secondary, university); self-reported level of physical activity (‘How frequently do you exercise?’: almost every day; two to three times a week, once a week, less than once a week, or never). Assessments included height, weight and spirometry (FEV_1_ and FVC). Postbronchodilator values were used if available or else prebronchodilator values. Predicted spirometry values were calculated using the Global Lung Function Initiative 2012 reference.^27^

### Statistical analyses

Characteristics of parents and offspring were summarised as frequencies and percentages for categorical variables, and as mean with SD or median with range or IQR for continuous variables with normal or skewed distributions, respectively.

Associations with breathlessness were analysed using multilevel logistic regression with random intercepts (using the STATA command ‘meqrlogit’). All models accounted for clustering within study centres and between siblings. Associations were presented as OR with 95% CI. Associations between covariates and breathlessness were first analysed separately for parents and offspring. The main analysis was of the association between breathlessness in parents (exposure of main interest) and breathlessness in their offspring (outcome), adjusted for potential confounders on two levels (parents and offspring).

Potential confounders were selected a priori based on subject matter knowledge and previous population studies^1–3 11–13 28^ using a directed acyclical graph (shown in online supplemental figure S1).^29^ Factors included were parental age, sex, Body Mass Index (BMI), asthma, current smoking, pack years of smoking, depression, education level, airflow obstruction (FEV_1_/FVC <0.70) and FVC. The primary model adjusted for factors measured in the parents, as these exposures preceded the outcome (offspring breathlessness). Models adjusted also for the similar factors in offspring were also fitted. We evaluated whether the association between parental and offspring breathlessness was modified by the sex of the parent and/or offspring by adding interaction terms to the fully adjusted model.

10.1136/thoraxjnl-2021-217271.supp1 Supplementary data

Sensitivity analyses were conducted: (1) adjusting for level of physical exercise in parents and offspring in addition to the other factors in the model. Physical activity was not included in the main model as it could be both a potential mediator or consequence (collider factor) rather than a determinant of breathlessness and thus potentially bias the findings; (2) not using the criterion ‘breathlessness when having wheezing and whistling in the chest at any time in the last 12 months in the outcome definition of breathlessness, due the its potential relationship to asthma; and (3) also adjusting for offspring lung function (level of airflow limitation (FEV_1_/FVC) and FVC) in the subcohort with data available; (4) analysing the association for parental breathlessness measured using the mMRC scale (available in parents only).^4^

Statistical significance was defined as a two-tailed p value of <0.05. All the analyses were conducted using STATA V.16.0.

## Results

A total of 1720 parents (mean age at inclusion 36 years (SD 6.4), 55% mothers) and 2476 offspring (mean age 30 years (SD 7.7), 55% daughters) were included in the analysis (table 1). Breathlessness was reported by 809 (32.7%) of parents and by 363 (14.7%) of offspring. Characteristics of participants by the presence of offspring breathlessness are shown in table 1.

**Table 1:** Characteristics of offspring (n=2476) and parents (n=1720) by the presence of offspring breathlessness

Factor	Offspring without breathlessness	Offspring with breathlessness	P value
Offspring factors			
N	2113	363	
Age, mean (SD)	29.8 (7.7)	30.5 (7.4)	0.074
Daughter	1130 (53.5%)	223 (61.4%)	0.005
Asthma	285 (13.6%)	198 (54.8%)	<0.001
BMI, mean (SD)	24.4 (4.5)	25.5 (5.3)	<0.001
BMI category (kg/m^2^)			<0.001
<20	246 (11.6%)	30 (8.3%)	
20–<25	1070 (50.6%)	159 (43.8%)	
25–<30	533 (25.2%)	103 (28.4%)	
≥30	199 (9.4%)	57 (15.7%)	
Missing	65 (3.1%)	14 (3.9%)	
Current smoker	242 (11.6%)	77 (21.6%)	<0.001
Pack-years of smoking, median (IQR)	0 (0–0.1)	0 (0–3.0)	<0.001
Depression	72 (10.0%)	33 (26.6%)	<0.001
Exercise level			0.035
Almost every day	466 (22.1%)	64 (17.6%)	
2–3 times a week	844 (39.9%)	130 (35.8%)	
Once a week	330 (15.6%)	67 (18.5%)	
Less than once a week	306 (14.5%)	65 (17.9%)	
Never	125 (5.9%)	29 (8.0%)	
Missing	42 (2.0%)	8 (2.2%)	
FEV_1_, mean (SD)	4.0 (0.8)	3.7 (0.8)	<0.001
FVC, mean (SD)	4.7 (1.0)	4.5 (1.0)	0.022
FEV_1_/FVC, mean (SD)	0.8 (0.1)	0.8 (0.1)	0.002
Parent factors			
Age, mean (SD)	35.7 (6.2)	36.5 (5.8)	0.021
Mother	1121 (53.1%)	210 (57.9%)	0.090
Breathlessness	651 (30.8%)	158 (43.5%)	<0.001
mMRC breathlessness score			0.008
0	1789 (84.7%)	286 (78.8%)	
1	246 (11.6%)	51 (14.0%)	
≥2	76 (3.6%)	24 (6.6%)	
Missing	2 (0.1%)	2 (0.6%)	
Asthma	302 (15.8%)	71 (23.4%)	0.001
Depression	190 (14.2%)	47 (18.7%)	0.069
BMI, mean (SD)	24.7 (4.3)	25.3 (4.6)	0.016
BMI category (kg/m^2^)			0.064
<20	155 (7.3%)	19 (5.2%)	
20–<25	1058 (50.1%)	165 (45.5%)	
25–<<30	618 (29.2%)	123 (33.9%)	
≥30	185 (8.8%)	40 (11.0%)	
Missing	97 (4.6%)	16 (4.4%)	
FEV_1_, mean (SD)	3.7 (0.8)	3.5 (0.7)	<0.001
FEV_1_ % of predicted, mean (SD)	112.4 (19.0)	107.2 (19.6)	<0.001
FVC, mean (SD)	4.6 (1.0)	4.4 (1.0)	0.006
FVC % of predicted, mean (SD)	110.1 (15.4)	108.6 (16.9)	0.17
FEV_1_/FVC, mean (SD)	0.8 (0.1)	0.8 (0.1)	<0.001
FEV_1_/FVC<0.7	114 (5.6%)	33 (9.5%)	0.006
Current smoker	667 (34.5%)	121 (37.8%)	0.25
Pack-years of smoking, median (IQR)	0 (0–12.6)	1.5 (0–15.5)	0.070
Highest education			0.11
Primary	187 (8.8%)	40 (11.0%)	
Secondary	651 (30.8%)	105 (28.9%)	
University	748 (35.4%)	105 (28.9%)	
Missing	527 (24.9%)	113 (31.1%)	
Exercise level			0.033
Every day	135 (6.4%)	26 (7.2%)	
4–6 times a week	400 (18.9%)	59 (16.3%)	
2–3 times a week	599 (28.3%)	88 (24.2%)	
Once a week	167 (7.9%)	22 (6.1%)	
Once a month	128 (6.1%)	29 (8.0%)	
Less than once a month	128 (6.1%)	24 (6.6%)	
Never	258 (12.2%)	64 (17.6%)	
Missing	298 (14.1%)	51 (14.0%)	

Data presented as frequency (%) or mean (SD) unless otherwise indicated.

BMI, Body Mass Index; mMRC, modified Medical Research Council.

Asthma was strongly related to breathlessness, both among parents and offspring (table 1). Asthma was more common in people with breathlessness versus those without breathlessness, both in parents (36.7% vs 4.5%, p<0.001) and in the offspring (54.6% vs 13.5%, p<0.001). Thus, about half of offspring with breathlessness also reported having asthma.

In parents, factors independently associated with higher breathlessness prevalence were male sex, obesity, asthma (strong association with adjusted OR 16.3, 95% CI 10.1 to 26.1), current smoking, history of depression, presence of airflow obstruction and lower FVC (table 2).

**Table 2:** Factors associated with breathlessness in parents

Parental factor	Unadjusted OR (95% CI)	Adjusted OR (95% CI)
Age (per 1 year)	1.0 (1.0 to 1.0)	1.0 (0.9 to 1.0)
Female versus male	0.9 (0.8 to 1.1)	0.5 (0.3 to 0.8)
Body Mass Index (kg/m^2^)		
<20	1.0 (0.7 to 1.4)	1.2 (0.6 to 2.3)
20–<25	1 (ref)	1 (ref)
25–<30	1.2 (1.0 to 1.4)	1.5 (0.7 to 2.9)
≥30	1.7 (1.3 to 2.39)	2.3 (1.0 to 5.1)
Asthma	15.1 (11.1 to 20.4)	16.3 (10.1 to 26.1)
Current smoker	1.9 (1.6 to 2.4)	2.0 (1.3 to 2.9)
Pack-year of smoking (per one unit)	1.0 (1.0 to 1.0)	1.0 (1.0 to 1.0)
Depression	2.1 (1.6 to 2.8)	1.8 (1.2 to 2.7)
Highest education		
Primary	1.3 (0.9 to 1.8)	1.1 (0.6 to 1.8)
Secondary	1 (ref)	1 (ref)
University	1.1 (0.9 to 1.3)	1.3 (1.0 to 1.9)
FEV_1_/FVC<0.7	4.7 (3.3 to 6.9)	3.5 (1.5 to 8.3)
FVC (per 1 L)	0.9 (0.8 to 1.0)	0.7 (0.5 to 0.8)

Associations analysed using random effects logistic regression accounting for clustering by study centre. Unadjusted analyses were conducted for each factor separately. In the adjusted model, each factor was adjusted for all other factors in the model.

ref, reference.

In offspring, a similar pattern of associations was found as that in parents (table 3), except that the sex distribution was reversed with greater breathlessness in women. Asthma was the factor most strongly related to breathlessness also in the offspring (OR 7.4, 95% CI 5.7 to 9.7). In these two samples of relatively young adults, age was not associated with breathlessness, neither in parents nor offspring.

**Table 3:** Factors associated with breathlessness in offspring

Offspring factor	Unadjusted OR (95% CI)	Adjusted OR (95% CI)
Age (per 1 year)	1.0 (1.0 to 1.0)	1.0 (1.0 to 1.0)
Female versus male	1.4 (1.1 to 1.7)	1.5 (1.1 to 2.0)
Body Mass Index (kg/m^2^)		
<20	0.8 (0.5 to 1.2)	0.7 (0.4 to 1.1)
20–<25	1 (ref)	1 (ref)
25–<30	1.4 (1.0 to 1.8)	1.4 (1.0 to 1.9)
≥30	2.1 (1.5 to 2.9)	2.0 (1.3 to 3.1)
Asthma	7.7 (6.0 to 9.8)	7.4 (5.7 to 9.7)
Current smoking	2.1 (1.6 to 2.8)	2.1 (1.5 to 3.1)
Pack-year of smoking (per one unit)	1.1 (1.0 to 1.1)	1.1 (1.0 to 1.1)
Depression	3.2 (2.0 to 5.2)	–
Highest education		
Primary	1.8 (1.0 to 3.5)	1.2 (0.6 to 2.6)
Secondary	1 (ref)	1 (ref)
University	1.2 (0.9 to 1.5)	1.0 (0.8 to 1.4)

Associations analysed using random effects logistic regression accounting for clustering by study centre. Unadjusted analyses were conducted for each factor separately. In the adjusted model, each factor was adjusted for all other factors in the mode. Due to high number of missing data, depression (n=1632 missing) and lung function (n=1381 missing) were not included in the multivariable model. When analysed in the subsample with data (in the fully adjusted model), depression associated with breathlessness (OR 2.2, 95% CI 1.2 to 3.9), whereas there were no clear associations for offspring airflow obstruction (FEV_1_/FVC<0.7, p=0.18, or FVC, p=0.55).

ref, reference.

In the two-generation analyses shown in table 4, parents with breathlessness were more likely to have offspring with breathlessness (19.5% vs 12.3% of their offspring reporting breathlessness, p<0.001; crude OR 1.7, 95% CI 1.3 to 2.2). In multivariable analysis, parents with breathlessness had independently increased risks of offspring reporting breathlessness. The association remained unchanged even after adjusting for both parental and offspring factors including asthma (adjusted OR 1.8, 95% CI 1.1 to 2.9) (table 4). In the sensitivity analysis excluding breathlessness in relation to wheeze from the outcome definition (as this was most likely related to asthma), the association between parental and offspring breathlessness was somewhat weaker (OR 1.5, 95% CI 0.7 to 3.2), and the precision decreased (and fewer participants and outcome events were included in the analyses) as shown by the wider CI in table 4. When also adjusting for offspring lung function (n=1381 (56%) with data) in addition to the other parental and offspring factors, parental breathlessness was still strongly associated with increased breathlessness in offspring (adjusted OR 2.4, 95% CI 1.1 to 5.3).

**Table 4:** Associations between breathlessness in parents and their offspring

Exposure variable	Association with offspring breathlessness, OR (95% CI)		
	Crude	Adjusted for parental factors only	Adjusted for both parental and offspring factors
Parental breathlessness, main analysis	1.7 (1.3 to 2.2) n=2476	1.8 (1.1 to 2.7) n=1143	1.8 (1.1 to 2.9) n=1040
Parental breathlessness, defined without concurrent wheeze	1.6 (1.1 to 2.5) n=2476	1.8 (0.9 to 3.4) n=1058	1.5 (0.7 to 3.2) n=961

Association between parental and offspring breathlessness, analysed using logistic regression, accounting for clustering within centres and between siblings. Estimates are expressed as ORs with 95% CIs. Adjustment factors in parents and offspring were age, sex, Body Mass Index category, asthma, current smoking, pack-years of smoking, depression (parents only), highest education, airflow limitation (FEV_1_/FVC<0.7, parents only) and FVC (parents only). See table 1 for abbreviations.

The association between parental breathlessness and higher risk of breathlessness in offspring was consistent when defining parental breathlessness using the mMRC scale. Compared with those with mMRC=0, parents with a mMRC of ≥2 had a more than three times increased odds of having offspring with breathlessness (OR 3.2, 95% CI 1.4 to 7.5), adjusting for both parental and offspring factors.

**Figure 1:**
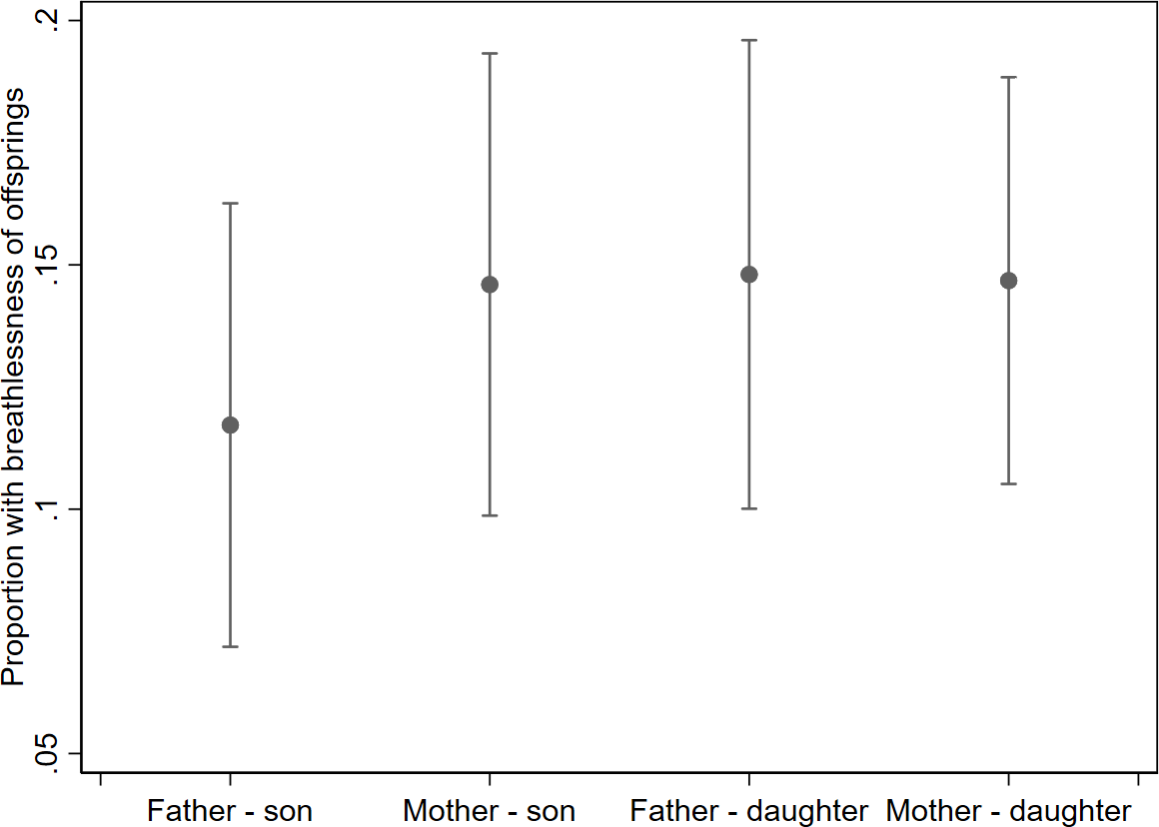
Breathlessness prevalence in offspring by sex of parent and child, adjusted for age of both generations. Analysed using random effects logistic regression accounting for clustering between siblings and within study centres. The model is fully adjusted for parental factors in table 2.

Female sex of either the parent (mother with breathlessness) or offspring (daughter) was associated with slightly higher prevalence of offspring breathlessness, as shown in figure 1. However, the sex of parent or offspring did not modify the association between parental and offspring breathlessness (p for interaction=0.48 by likelihood ratio test). The association between parental and offspring breathlessness was similar when adjusting also for physical exercise of parents and offspring in addition to the other factors in the model (OR 1.8, 95% CI 1.1 to 3.0).

## Discussion

In this large international study of 1720 parents and 2476 offspring, there was substantial clustering of breathlessness across two generations. Parents who reported breathlessness as young adults were more likely to have offspring who had developed breathlessness as young adults. Obesity, smoking, depression, asthma, lower lung function and female sex were associated with breathlessness in each generation. If the parents had breathlessness, the odds of having an offspring with breathlessness was increased by 80%, even after controlling for these determinants of breathlessness in both the parents and the offspring.

This is the first study exploring breathlessness across generations. The findings extend the understanding of breathlessness in several important ways. First, it finds that factors associated with breathlessness were similar in both parents and their offspring, which is a novel finding. The expected associations with increased breathlessness were seen for factors such as obesity, airflow obstruction and FVC, which supports the validity of the present findings. In a novel second step, we reported a strong association between parental and offspring breathlessness. Third, this clustering of breathlessness across the generations was not explained by a range of the known risk factors for breathlessness in the population.^1–3 11–13 28^ Finally, this association did not show clear sex-specific patterns, although a weaker association was indicated for the father-son relationships.

Heredity of breathlessness, a multifactorial and multidimensional symptom,^10^ likely involves a complex interplay of social, behavioural and other environmental, as well as genetic and possibly epigenetic factors. Genetic factors have been implicated in the development of airflow limitation, reduced lung function and impaired ventilatory capacity,^21^ which are important determinants of breathlessness.^13–15^ Lung function and respiratory symptoms could also be influenced by early life and environmental exposures.^9 22^ Genetic factors predict increased risk of developing chronic airflow limitation, independent of smoking status,^21^ and influence the clinical phenotype and functional impairment in COPD.^30^ Other partly inheritable factors include BMI and body fat distribution,^31^ and level of physical activity and sedentary behaviour.^23^

Genetic heritability has also been shown for anxiety and panic, including increased anxiety response to hypercapnia in a twin study.^32^ Mood including anxiety and depression is strongly related to breathlessness perception, and clustering of breathlessness across generations might be influenced by inheritable personality traits.^33^ In a study of pain, the symptom phenotype had significant and positive genetic correlations with depressive symptoms, major depressive disorders and neuroticism, and all pain phenotypes were heritable.^34^ Similar genetic studies of breathlessness are lacking.

Asthma is a likely heritable cause of breathlessness across generations, especially in younger people who have a relatively high prevalence of asthma compared with that of other chronic conditions causing breathlessness.^16^ Asthma has substantial genetic components,^18^ as demonstrated in twin studies.^19^ The cross-generational association in the present study became somewhat weaker when excluding concurrent wheeze in the breathlessness definition, indicating that (undiagnosed) asthma could be a factor underpinning the present heredity pattern for breathlessness. Hereditability was supported as the association remained in the most fully adjusted model which accounted also for lung function of both generations.

Strengths of this study include the use of a large, well characterised, multicentre database. The analysis accounted for clustering within centres and among siblings and evaluated a range of relevant factors in parents and their offspring.

A limitation was the definition of breathlessness. Optimally, breathlessness should be measured using validated instruments at standardised level of physical activity,^35^ but such measurements are rarely available in epidemiological and large-scale studies or across generations. We used available data on breathlessness, using a similar definition in parents and offspring. Findings were similar when analysing breathlessness using mMRC (available in parents only) and in a sensitivity analysis excluding concurrent wheeze. The breathlessness questions used may relate to varying extent to asthma, and the persistent strong association after adjusting for covariates including a history of asthma might reflect undiagnosed disease.

Although the analyses controlled for a range of relevant clinical factors including lung function, data were unavailable on a number of potential determinants of breathlessness including heart function and physical fitness, which should be further explored across generations. Another potential limitation is that many invited parents and offspring did not participate in the study. A previous analysis of the influence of missing data in the Respiratory Health in Northern Europe study showed that participants tended to be healthier, but that exposure–outcome associations remained unchanged by this selection.^36^ Based on this, while the prevalence of factors may be underestimated (including breathlessness), the analysis estimates are likely to be valid.

We propose further research to validate of the present findings using standardised measurement of multidimensional breathlessness; evaluate the influence of anxiety, psychosocial factors including personality traits, and genetic as well as epigenetic variations. This research is important for improved knowledge on the mechanisms underpinning breathlessness across the generations, to identify risk groups for developing severe breathlessness and to identify environmental factors amenable to interventions to improve respiratory health of coming generations.

In conclusion, people with breathlessness were more likely to have children who reported breathlessness as young adults. The clustering of breathlessness across generations was independent of lung function, asthma and a range of potential confounders in both generations and warrants further investigation.

10.1136/thoraxjnl-2021-217271.supp2 Supplementary data

## Data Availability

Data are available upon reasonable request. Deidentified data underlying the analyses are available upon reasonable request to the Respiratory Health in Northern Europe, Spain and Australia generation study (email: postmottak@helse-bergen.no).
