# Supplementary material for: Breathlessness across generations: results from the RHINESSA generation study
Source: Thorax. 2021 Jun 14;77(2):172–7. doi: 10.1136/thoraxjnl-2021-217271 (PMC8762033; doi:10.1136/thoraxjnl-2021-217271)

Supplement

Breathlessness across generations: results from the RHINESSA generation study

Figure S1. Directed acyclical graph (DAG) of the effect between parental and offspring breathlessness.

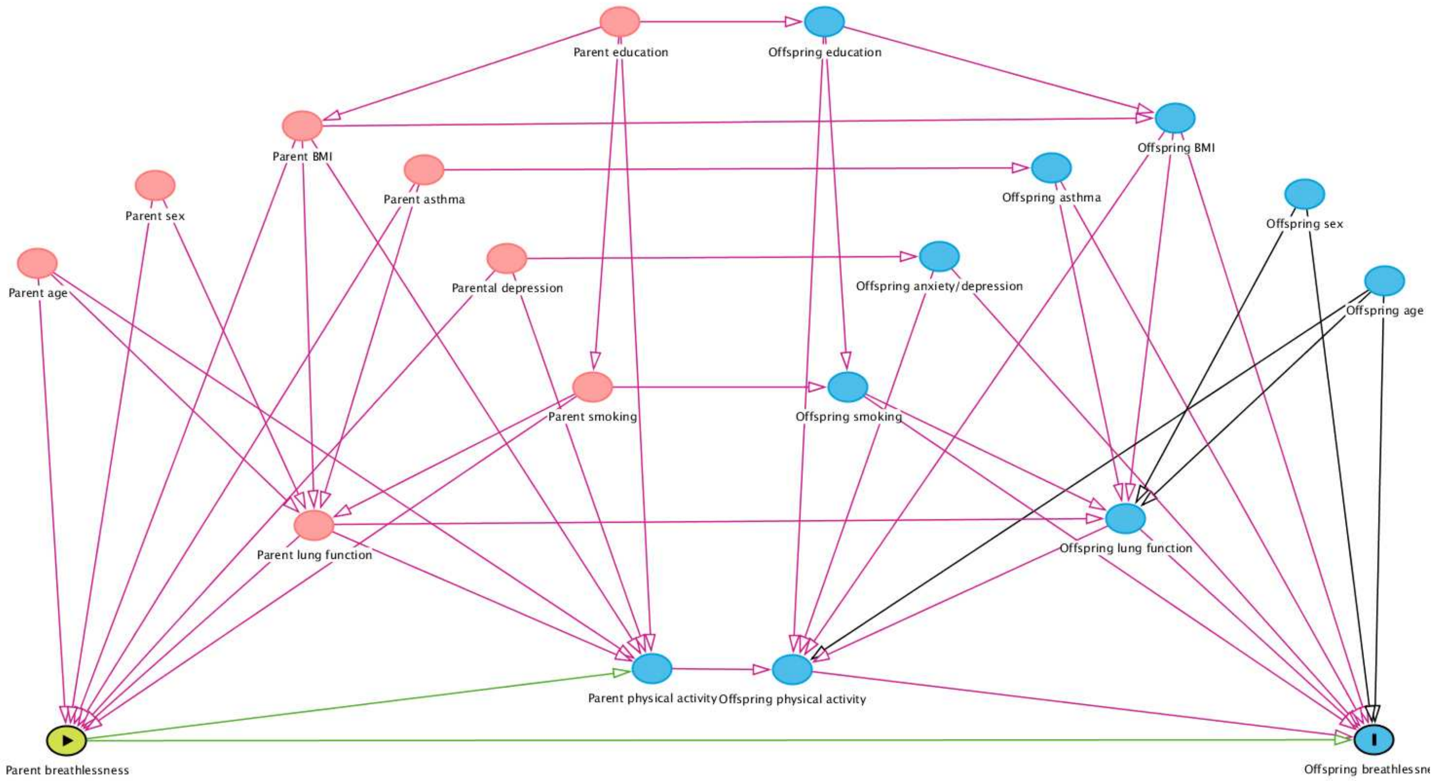

Supplement: Supplementary data [file thoraxjnl-2021-217271supp001.pdf]
