# Supplementary material for: Breathlessness across generations: results from the RHINESSA generation study
Source: Thorax. 2021 Jun 14;77(2):172–7. doi: 10.1136/thoraxjnl-2021-217271 (PMC8762033; doi:10.1136/thoraxjnl-2021-217271)
Supplement: Supplementary data [file thoraxjnl-2021-217271supp002.pdf]

Overview of ethics committees and approval numbers of RHINESSA centers

Study center/ country	Name of ethics committee	Transcript/ approval number
Sweden (multicenter: Uppsala, Umeå and Göteborg) screening	Regional Ethical Review Board in Uppsala	Dnr 2013/ 352
Sweden (multicenter: Uppsala, Umeå and Göteborg) clinical	Regional Ethical Review Board in Uppsala	Dnr 2016/ 023
Melbourne, Australia	Alfred Hospital Human Research Ethics Committee	HREC/ 17/Alfred/ 144
Iceland, Reykjavik questionnaire	The National Bioethics Committee	VSN-13-190
Iceland, Reykjavik clinical	The National Bioethics Committee	VSN-16-070
Denmark	Ethical Scientific Committee for Mid Region Jylland, Denmark	1-10-72-301-15
Spain, Huelva	Comité de ética de la investigación de la provincia de Huelva(Research Ethics Committee of the Province of Huelva)	Approval date April 4, 2013
Spain, Albacete	Comité ético de investigación clínica del Complejo Hospitalatio Universitario de Albacete. (Ethic Committee of clinical Research of the University Hospital Complex of Albacete)	Institutional Review Board IRB 00006998
Estonia, Tartu	Research Ethics Committee of the University of Tartu (UT REC)	233/ T-7
Norway, Bergen	Regional Committee of Medical and Health Research Ethics, Rec West	2012/1077
